# Epidemic Keratoconjunctivitis-Causing Adenoviruses Induce MUC16 Ectodomain Release To Infect Ocular Surface Epithelial Cells

**DOI:** 10.1128/mSphere.00112-15

**Published:** 2016-02-10

**Authors:** Balaraj B. Menon, Xiaohong Zhou, Sandra Spurr-Michaud, Jaya Rajaiya, James Chodosh, Ilene K. Gipson

**Affiliations:** a Schepens Eye Research Institute, Harvard Medical School, Boston, Massachusetts, USA; b Massachusetts Eye and Ear Infirmary, Harvard Medical School, Boston, Massachusetts, USA; c Department of Ophthalmology, Harvard Medical School, Boston, Massachusetts, USA; Boston University School of Medicine

**Keywords:** adenoviruses, keratoconjunctivitis, mucin

## Abstract

Human adenoviruses (HAdV), species D in particular (HAdV-D), are frequently associated with epidemic keratoconjunctivitis (EKC). Although the infection originates at the ocular surface epithelium, the mechanisms by which HAdV-Ds bypass the membrane-associated mucin (MAM)-rich glycocalyx of the ocular surface epithelium to trigger infection and inflammation remain unknown. Here, we report that an EKC-causing adenovirus (HAdV-D37), but not a non-EKC-causing one (HAdV-D19p), induces ectodomain release of MUC16—a MAM with barrier functions at the ocular surface—from cultured human corneal and conjunctival epithelial cells. HAdV-D37, but not HAdV-D19p, is also found to decrease the glycocalyx barrier function of corneal epithelial cells, as determined by rose bengal dye penetrance assays. Furthermore, results from quantitative PCR (qPCR) amplification of viral genomic DNA using primers specific to a conserved region of the E1B gene show that, in comparison to infection by HAdV-D19p, infection by HAdV-D37 is significantly increased in corneal epithelial cells. Collectively, these results point to a MUC16 ectodomain release-dependent mechanism utilized by the EKC-causing HAdV-D37 to initiate infection at the ocular surface. These findings are important in terms of understanding the pathogenesis of adenoviral keratoconjunctivitis. Similar MAM ectodomain release mechanisms may be prevalent across other mucosal epithelia in the body (e.g., the airway epithelium) that are prone to adenoviral infection.

**IMPORTANCE** Human adenoviruses (HAdVs) are double-stranded DNA viruses that cause infections across all mucosal tissues in the body. At the ocular surface, HAdVs cause keratoconjunctivitis (E. Ford, K. E. Nelson, and D. Warren, Epidemiol Rev 9:244–261, 1987, and C. M. Robinson, D. Seto, M. S. Jones, D. W. Dyer, and J. Chodosh, Infect Genet Evol 11:1208–1217, 2011, doi:10.1016/j.meegid.2011.04.031)—a highly contagious infection that accounts for nearly 60% of conjunctivitis cases in the United States (R. P. Sambursky, N. Fram, and E. J. Cohen, Optometry 78:236–239, 2007, doi:10.1016/j.optm.2006.11.012, and A. M. Pihos, J Optom 6:69–74, 2013, doi:10.1016/j.optom.2012.08.003). The infection begins with HAdV entry within ocular surface epithelial cells; however, the mechanisms used by HAdVs to transit the otherwise protective mucosal barrier of ocular surface epithelial cells prior to entry remain unknown. Here, we report that the highly virulent keratoconjunctivitis-causing HAdV-D37 induces release of the extracellular domain (ectodomain) of MUC16, a major component of the mucosal barrier of ocular surface epithelial cells, prior to infecting underlying cells. Currently, there is no specific treatment for controlling this infection. Understanding the early steps involved in the pathogenesis of keratoconjunctivitis and using this information to intercept adenoviral entry within cells may guide the development of novel strategies for controlling the infection.

## Abstract (precis)

Human adenoviruses (HAdVs) are double-stranded DNA viruses that cause infections across all mucosal tissues in the body. At the ocular surface, HAdVs cause keratoconjunctivitis (E. Ford, K. E. Nelson, and D. Warren, Epidemiol Rev 9:244–261, 1987, and C. M. Robinson, D. Seto, M. S. Jones, D. W. Dyer, and J. Chodosh, Infect Genet Evol 11:1208–1217, 2011, doi:10.1016/j.meegid.2011.04.031)—a highly contagious infection that accounts for nearly 60% of conjunctivitis cases in the United States (R. P. Sambursky, N. Fram, and E. J. Cohen, Optometry 78:236–239, 2007, doi:10.1016/j.optm.2006.11.012, and A. M. Pihos, J Optom 6:69–74, 2013, doi:10.1016/j.optom.2012.08.003). The infection begins with HAdV entry within ocular surface epithelial cells; however, the mechanisms used by HAdVs to transit the otherwise protective mucosal barrier of ocular surface epithelial cells prior to entry remain unknown. Here, we report that the highly virulent keratoconjunctivitis-causing HAdV-D37 induces release of the extracellular domain (ectodomain) of MUC16, a major component of the mucosal barrier of ocular surface epithelial cells, prior to infecting underlying cells. Currently, there is no specific treatment for controlling this infection. Understanding the early steps involved in the pathogenesis of keratoconjunctivitis and using this information to intercept adenoviral entry within cells may guide the development of novel strategies for controlling the infection.

## OBSERVATION

Adenoviruses (HAdVs) are nonenveloped, icosahedral particles with a proteinaceous capsid that encapsulates a double-stranded DNA genome. To date, more than 70 human-specific types have been recognized. These HAdVs, which can be divided into seven species (HAdV-A to -G), are capable of causing infections of the ocular surface and respiratory, gastrointestinal, and genitourinary tracts (1). However, only those belonging to species D (HAdV-D) are commonly associated with epidemic keratoconjunctivitis (EKC) (2, 3). The clinical manifestation of this infection is the development of severe membranous conjunctivitis and epithelial keratitis, followed by multifocal subepithelial infiltrates in the stroma that cause photophobia and reduced vision (4). The stromal infiltrates usually develop within 7 to 10 days after onset of the clinical signs of infection and may persist for months to years (5, 6).

Interestingly, not all HAdV-Ds are associated with keratoconjunctivitis. For instance, HAdV-D37 causes EKC, whereas HAdV-D19p does not. A single amino acid, Lys^240^, in the fiber knob domain of HAdV-D37 was found to be crucial in determining its binding to conjunctival epithelial cells (7). The ocular tropism exhibited by HAdV-Ds is also thought to be related to the expression of specific receptors at the ocular surface. HAdV-Ds are thought to use the GD1a glycan (8), sialic acid (9, 10), and possibly CD46 (1) as cellular receptors, rather than the prototypical coxsackievirus and adenovirus receptor (CAR).

In recent years, advancements have been made in identifying HAdV-specific receptors on epithelial surfaces and mechanisms that promote apical entry of human adenoviruses into epithelial cells (11–13). In lung epithelial cells, chemotactic cytokines, such as interleukin 8 (IL-8), have been shown to trigger a signaling cascade that causes relocation of CAR and the αvβ3 integrin coreceptor to the apical surface, which promotes adenovirus binding and uptake (13). However, the mechanism(s) by which HAdVs traverse through the membrane-associated mucin (MAM)-rich glycocalyx that covers the apical surface of all mucosal epithelia in the body to gain access to receptors and initiate infection still remains unknown. The only data pertaining to MAM-HAdV interactions on mucosal epithelia come from studies performed in the airway epithelium. These studies have suggested that the MAM-rich glycocalyx is a barrier to adenoviral vectors and adenovirus-mediated gene transfer (14–16). A more recent study demonstrated that the MAM glycocalyx of human tracheobronchial epithelial cells restricts adenoviruses while permitting penetrance of the much smaller adeno-associated virus (17). Although this observation suggests that particle exclusion by the glycocalyx is, in part, size dependent, it also raises this question: how do infection-causing adenoviruses overcome the MAM glycocalyx on epithelial surfaces prior to triggering infection? We have begun to address this question using the ocular surface epithelium as a model system along with a highly virulent EKC-causing adenovirus, HAdV-D37.

At the ocular surface, invading pathogens first encounter the tear film. In addition to antimicrobial proteins, the tear film also consists of both secreted mucins and shed MAMs in the aqueous layer that move around and serve to trap and wash away pathogens and debris from the epithelial surface. Contrary to tear mucins, MAMs remain physically tethered to the apical surface of epithelial cells and, as such, constitute the cellular interface between invading pathogens and underlying epithelial cells. Thus, the ability of a pathogen to manipulate the MAM glycocalyx and gain access to underlying ocular surface epithelial cells determines the outcome of infection. The MAM repertoire of the ocular surface epithelium, which is shared by the airway epithelium, primarily includes MUC1, MUC4, and MUC16 (18). MUC1 and MUC16 are expressed by the corneal and conjunctival epithelia, while MUC4 is expressed by the latter (18, 19). Several lines of investigation have indicated that MUC16 is the major contributor of barrier function at the ocular surface (20, 21). MUC16 not only prevents the bacterium *Staphylococcus aureus* from adhering to and invading human corneal epithelial cells (20, 21) but also contributes to the maintenance of immune homeostasis (22). Furthermore, O-glycans within the N-terminal portion of the molecule’s ectodomain are also known to contribute to barrier function (23, 24). Given these protective roles of this MAM, we hypothesized that EKC-causing HAdV-Ds utilize a MUC16 barrier-disrupting mechanism to infect underlying ocular surface epithelial cells. Previously, we reported that a conjunctivitis-causing, unencapsulated strain of *Streptococcus pneumoniae* secretes an extracellular zinc metalloproteinase (ZmpC) to cleave the ectodomain of MUC16 and infect ocular surface epithelial cells (25). However, such a mechanism cannot be envisioned for EKC-causing HAdV-Ds because, unlike bacteria, adenoviruses are inert entities and can begin synthesizing proteases only upon entry and replication within host cells.

**FIG 1:**
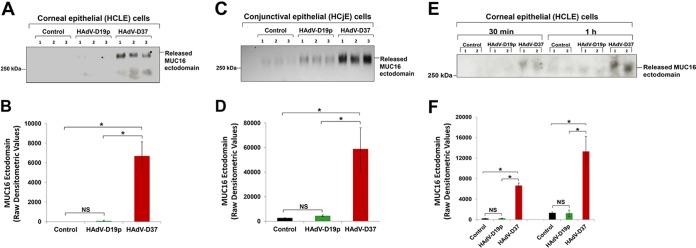
EKC-causing HAdV-D37 induces MUC16 ectodomain release from human corneal and conjunctival epithelial cells. (A and C) HCLE and HCjE cells were exposed to HAdV-D19p and HAdV-D37 at identical MOIs, following which equal volumes of culture supernatants were collected and analyzed for released MUC16 ectodomain. Control cells were not exposed to HAdV-Ds. The results shown are from experiments performed in biological triplicates. Identical patterns of MUC16 ectodomain release were observed in separate experiments. The faint bands observed in the “Control” condition in panel C are attributed to constitutive MUC16 ectodomain shedding (25). The precise mechanism of constitutive ectodomain shedding of MAMs remains unknown (20, 25, 45). (B and D) Bar graphs represent band intensities (raw densitometric values) corresponding to the MUC16 ectodomain in the blots shown in panels A and C above. (E) HCLE cells were exposed to HAdV-D19p and HAdV-D37 at identical MOIs for 30 min and 1 h, following which MUC16 ectodomain release was analyzed. The results shown represent experiments performed in biological duplicates. (F) Graph representing band intensities (raw densitometric values) corresponding to the MUC16 ectodomain in the blot shown in panel E. *, *P* < 0.05, Bonferroni test; NS, not significant.

To test our hypothesis, human corneal-limbal epithelial (HCLE) and conjunctival epithelial (HCjE) cells, cultured for optimal mucin expression, were incubated with the EKC-causing HAdV-D37 and non-EKC-causing HAdV-D19p at a multiplicity of infection (MOI) of 3 for 2 h. Following incubation, equal volumes of culture supernatants were collected, concentrated, and analyzed for released MUC16 ectodomain by Western blotting. Surprisingly, HAdV-D37, but not HAdV-D19p, was found to induce the release of MUC16 ectodomain from differentiated HCLE and HCjE cells (Fig. 1A to D). Neither HAdV-D37 nor HAdV-D19p induced MUC1 ectodomain release (data not shown). Additionally, MUC16 ectodomain release from HCLE cells was observed as early as 30 min after exposure to HAdV-D37 (Fig. 1E and F), which suggests that the ectodomain release process likely occurs prior to HAdV-D37 entry within epithelial cells.

**FIG 2:**
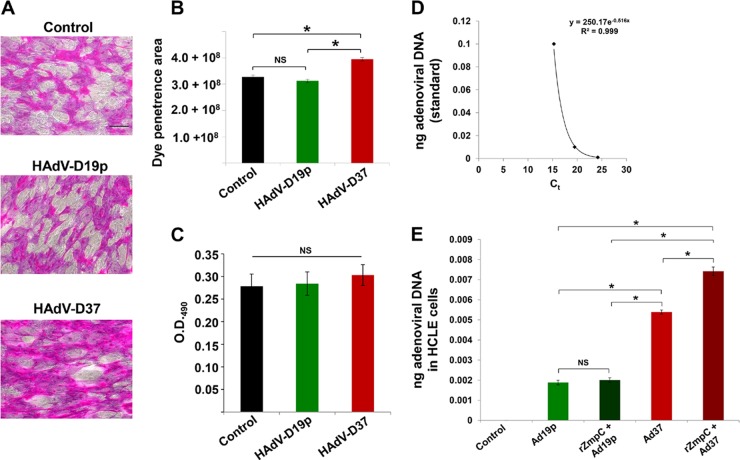
EKC-causing HAdV-D37 decreases glycocalyx barrier function and exhibits increased infectivity of corneal epithelial cells. (A) Representative micrographs of HCLE cells exposed to HAdV-D19p and HAdV-D37 for 2 h and later incubated with rose bengal dye are shown. Control cells were not exposed to HAdV-Ds. Scale bar = 50 µm. (B) Quantitative analyses (*n* = 30 for each condition) of areas of cells exhibiting rose bengal dye penetrance. *, *P* < 0.05, Bonferroni test; NS, not significant. (C) Values for optical density at 490 nm (OD_490_), corresponding to the levels of LDH released into the culture supernatants under the conditions described in the legend to panel A. NS, not significant at a *P* value of <0.05 by Kruskal-Wallis test. (D) Standard curve generated by plotting the cycle threshold (C_t_) required to amplify the E1B gene from known amounts of adenoviral DNA (0.1 ng, 0.01 ng, and 0.001 ng) by qPCR. (E) Quantification of adenoviral DNA recovered from untreated and rZmpC-pretreated HCLE cells that were exposed to HAdV-D19p and HAdV-D37 at identical MOIs for 2 days. The equation resulting from the standard curve shown in panel D was used to quantify adenoviral DNA in HCLE cells. *, *P* < 0.05, Bonferroni test; NS, not significant.

To determine whether the glycocalyx barrier function of corneal epithelial cells is affected upon exposure to HAdV-D37, rose bengal dye penetrance assays were performed. This assay is a well-established method for determining the health of ocular surface epithelial cells (20, 25, 26) and relies on the extent to which the dye penetrates epithelial cells. Typically, healthy, fully differentiated corneal epithelial cells exclude the dye; however, under conditions of reduced MUC16 expression (e.g., in confluent or undifferentiated cells), increased dye penetrance is observed (20, 21, 25). In this study, incubation of differentiated HCLE cells with HAdV-D37 at an MOI of 3 for 2 h resulted in a significant increase in rose bengal dye penetrance compared to the levels of penetrance under the HAdV-D19p incubation and control conditions (Fig. 2A and B). No difference in dye penetrance was observed between the HAdV-D19p incubation and control conditions (Fig. 2A and B). Furthermore, to determine whether the increased dye penetrance observed under the HAdV-D37 incubation condition could be a result of cell death and loss of apical MUC16-expressing epithelial cells, cytotoxicity assays were performed by measuring the levels of lactate dehydrogenase (LDH) released into the culture supernatants (27). The results from this assay revealed no significant increase in LDH levels in the culture supernatants under HAdV-D37 and HAdV-D19p incubation and control conditions (Fig. 2C). These data suggest that HAdV-D37 exposure most likely compromises the glycocalyx barrier of corneal epithelial cells as a consequence of MUC16 ectodomain release and not due to cytotoxic effects.

Loss of the ectodomain of MUC16 from corneal epithelial cells has been previously shown to facilitate invasion by bacterial pathogens (20, 25). To determine whether HAdV-D37-induced MUC16 ectodomain release promotes HAdV-D37 infection of ocular surface epithelial cells, differentiated HCLE cells were incubated independently with HAdV-D37 and HAdV-D19p at an MOI of 3 for 2 days, following which adenoviral loads were measured by quantitative PCR (qPCR) amplification of a highly conserved region of the E1B gene (28) and values extrapolated from a standard curve (Fig. 2D). The qPCR data revealed increases of 2.5- to 3-fold in E1B copies in HAdV-D37-incubated cells compared to the levels under control and HAdV-D19p incubation conditions (Fig. 2E). Furthermore, HCLE cells pretreated with rZmpC—a recombinant pneumococcal zinc metalloproteinase known to cleave the ectodomain of MUC16 (25, 29)—showed a significant increase in infection by HAdV-D37 but not by HAdV-D19p (Fig. 2E). That rZmpC pretreatment of HCLE cells did not enhance infection by HAdV-D19p in comparison to its level of infection under the untreated condition may be explained, in part, by the inability of HAdV-D19p to bind to specific receptors on corneal epithelial cells. In fact, a recent study demonstrated that HAdV-D19p is unable to replicate within cultured corneal epithelial cells despite a 1-week exposure (28).

Taken together, these data demonstrate for the first time that a virulent adenovirus is capable of manipulating the MAM glycocalyx barrier of a mucosal epithelium to infect underlying cells. While the mechanism of HAdV-D37-induced MUC16 ectodomain release remains to be elucidated, one interesting hypothesis is that binding of HAdV-D37 to MUC16 induces self-cleavage of the MAM. Indeed, such a mechanism has been demonstrated for the MAM MUC1 (30). The exposure of gastric epithelial cells to bacterium-sized beads coated with antibodies to the ectodomain of MUC1 was found to induce release of the MAM’s ectodomain (30). This finding is important and suggests that binding of ligands to MAMs can induce release of their ectodomains. It will be interesting to determine whether HAdV-D37 interacts with MUC16, especially since HAdV-D37 is known to use sialic acid as a receptor for binding to corneal epithelial cells (9) and the ectodomain of MUC16 contains a terminal *O*-acetyl sialic acid moiety, also known as the H185 epitope (31). With regard to the site of cleavage within MUC16, the ectodomain of MUC16 has 56 SEA modules interspersed within the tandem repeat region of the protein (18, 32, 33), and the 55th and 56th SEA modules contain predicted proteolytic cleavage sites (34–36). Although the SEA module within MUC1 is known to be a self-cleaving domain (37), the significance of such a module in self-cleavage of MUC16 needs to be investigated.

The data from the rose bengal dye penetrance assay indicate that HAdV-D37 exposure compromises the glycocalyx barrier function of corneal epithelial cells. Since MUC16, with a molecular mass of >2.5 mDa, is the largest known MAM and has the potential to extend up to 250 to 300 nm from the apical cell surface (38), release of the ectodomain of MUC16 may facilitate the access of HAdV-D37 to its receptor(s) for subsequent internalization within epithelial cells. Another possibility is that release of the ectodomain of MUC16 somehow alters the function of tight junctions of corneal epithelial cells to expose HAdV-D37-specific receptors. Recently, it was demonstrated that MUC16 knockdown HCLE cells exhibit decreased tight junction function and disruption of the actin cytoskeleton (20). However, while MUC16 knockdown cells lack the cytoplasmic tail of the MAM, which contains an ezrin, radixin, and moeisin (ERM)-binding domain necessary for linking the ERM to actin (21), HAdV-D37-exposed cells most likely retain the cytoplasmic tail. Moreover, enzymatic cleavage of MUC16 by rZmpC does not result in loss of tight junctions or decreased transepithelial resistance (20, 25). Therefore, it does not appear that HAdV-D37-induced MUC16 ectodomain release interferes with tight junction function, at least during the first 2 h of incubation with the adenovirus. Rather, loss of the MUC16 barrier may serve as a mechanism to promote interaction of HAdV-D37 with its receptor(s) on the apical cell surface.

Clearly, follow-up experiments are needed to understand the mechanistic basis of interactions between HAdV-D37 and MUC16 at the ocular surface and to determine whether a similar MAM ectodomain release strategy is used by adenoviruses to trigger infections at other mucosal surfaces. It would also be ideal to corroborate the data using a mouse model. However, establishing a mouse model is not feasible because, (i) unlike humans, mice do not express MUC16 in the corneal epithelium (2, 39) and (ii) mice are considered to be poor models for HAdV replication (40, 41). Nevertheless, we believe that our *in vitro* data reflect some of the earliest molecular events that precede the establishment of adenoviral keratoconjunctivitis. From a translational standpoint, blockade of HAdV-D37-induced MUC16 ectodomain release may represent a novel approach for controlling the spread of adenoviral keratoconjunctivitis.

### Cell lines and culture methods.

Telomerase-transformed HCLE and HCjE cell lines for which mucin gene expression has been well characterized were used (42). These cell lines mimic several aspects of native ocular surface epithelial cells, especially MAM expression (42). HCLE and HCjE cells were cultured and grown to confluence in keratinocyte serum-free medium (K-SFM) (Invitrogen) containing 25 µg/ml bovine pituitary extract and 0.2 ng/ml epidermal growth factor (EGF). The cells were later switched to Dulbecco’s modified Eagle’s medium–nutrient mixture F-12 (DMEM–F-12) (Cellgro) supplemented with 10% calf serum and 10 ng/ml EGF for 7 days to promote differentiation and optimal MAM expression (42). Stratified HCLE and HCjE cells were washed three times with antibiotic- and EGF-free K-SFM prior to incubation with the HAdV-Ds diluted in the same medium.

### Adenoviruses.

Both HAdV-D19p and HAdV-D37 were obtained from the ATCC, propagated in A549 cells, and purified by cesium-chloride gradient centrifugation. Purified adenoviruses were dialyzed against dialysis buffer (10 mM Tris, 80 mM NaCl, 2 mM MgCl_2_, 10% glycerol), and their titers were determined in triplicate using A549 cells (28, 43).

### Western blot analysis to quantify MUC16 ectodomain release.

Western blotting to quantify MUC16 ectodomain release using culture supernatants derived from HAdV-D19p- and HAdV-D37-exposed HCLE and HCjE cells was performed as described previously (25, 29). Briefly, 500-µl-amounts of cell culture supernatants were collected from cultures incubated under each condition, concentrated using a 10-kDa-cutoff concentrator (Millipore), and separated by SDS-agarose electrophoresis. Western blotting to detect the ectodomain of MUC16 was done using the ectodomain-specific M11 antibody (44) (NeoMarkers) as the primary antibody and horseradish peroxidase-conjugated goat anti-mouse IgG1 (Santa Cruz Biotechnology) as the secondary antibody. The blots were developed using the SuperSignal West femto maximum sensitivity substrate (Thermo Scientific). The band intensities were analyzed using the ImageJ software from NIH.

### Rose bengal dye penetrance assay.

HCLE cells were exposed to HAdV-D19p and HAdV-D37 at an MOI of 3 for 2 h. Culture supernatants were then collected and saved for performing cytotoxicity assays, while the cells were rinsed with phosphate-buffered saline (PBS) and incubated with a 0.1% solution of rose bengal dye prepared in PBS. After a 5-min incubation, the dye was aspirated and 5 images per well (a total of six wells was used for each condition) were immediately photographed using a 10× objective on a Nikon inverted Eclipse TS100 microscope with a Spot Insight camera (Diagnostic Instruments, Inc.). The areas of dye penetration were quantified using the ImageJ software from NIH, as previously described (20, 21, 25).

### Cytotoxicity assay.

This assay was performed using the CytoTox 96 nonradioactive kit (Promega) following the manufacturer’s instructions. Fifty-microliter amounts of the culture supernatants that were collected prior to performing rose bengal dye penetrance assays were used in each reaction mixture. The amount of color developed in each well was read spectrophotometrically at an absorbance of 490 nm. A standard curve for this assay is included in Fig. S1 in the supplemental material.

10.1128/mSphere.00112-15.1 Figure S1  Standard curve for the LDH assay. (A) Defined numbers of HCLE cells, denoted on the *x* axis, were lysed by repeated freeze-thaw treatment. The LDH released into the supernatants was then measured using the CytoTox 96 nonradioactive kit. The resulting OD_490_ values are plotted on the *y* axis. (B) OD_490_ values within the linear range shown in panel A were replotted to obtain the slope from the equation. Download Figure S1, DOC file, 0.4 MB. Copyright © 2016 Menon et al. 2016 Menon et al. This content is distributed under the terms of the Creative Commons Attribution 4.0 International license.

### Adenoviral E1B gene quantification by qPCR.

Differentiated HCLE cells were exposed to HAdV-D19p and HAdV-D37 at an MOI of 3 for 2 days, following which cells were harvested and DNA extracted using the QIAamp DNA blood minikit (Qiagen). Under conditions involving pretreatment with rZmpC, HCLE cells were incubated with 200 pmol of the enzyme for 4 h prior to incubation with HAdV-Ds. qPCR amplification of viral genomic DNA was performed using primers specific to a highly conserved region of the E1B gene (forward primer, 5′ TGCTCTGGCCTGCTAGATTC 3′, and reverse primer, 5′ CTGGCTCCATTTGTCAACCAG 3′) as described previously (28), using RT^2^ SYBR green mastermix (Qiagen). qPCR was performed on an Eppendorf Mastercycler ep gradient S platform. Quantification of the E1B copies in HAdV-D19p- and HAdV-D37-exposed HCLE cells was extrapolated from a standard curve.

### Statistical analyses.

Statistical analyses were performed using one-way analysis of variance (ANOVA) to determine overall significance. Analyses were performed using the GraphPad InStat 3 program for Macintosh, version 3.1a. A *P* value of <0.05 was considered significant.
