# Supplementary figures and images for: Epidemic Keratoconjunctivitis-Causing Adenoviruses Induce MUC16 Ectodomain Release To Infect Ocular Surface Epithelial Cells
Source: mSphere. 2016 Feb 10;1(1):e00112-15. doi: 10.1128/mSphere.00112-15 (PMC4863608; doi:10.1128/mSphere.00112-15)

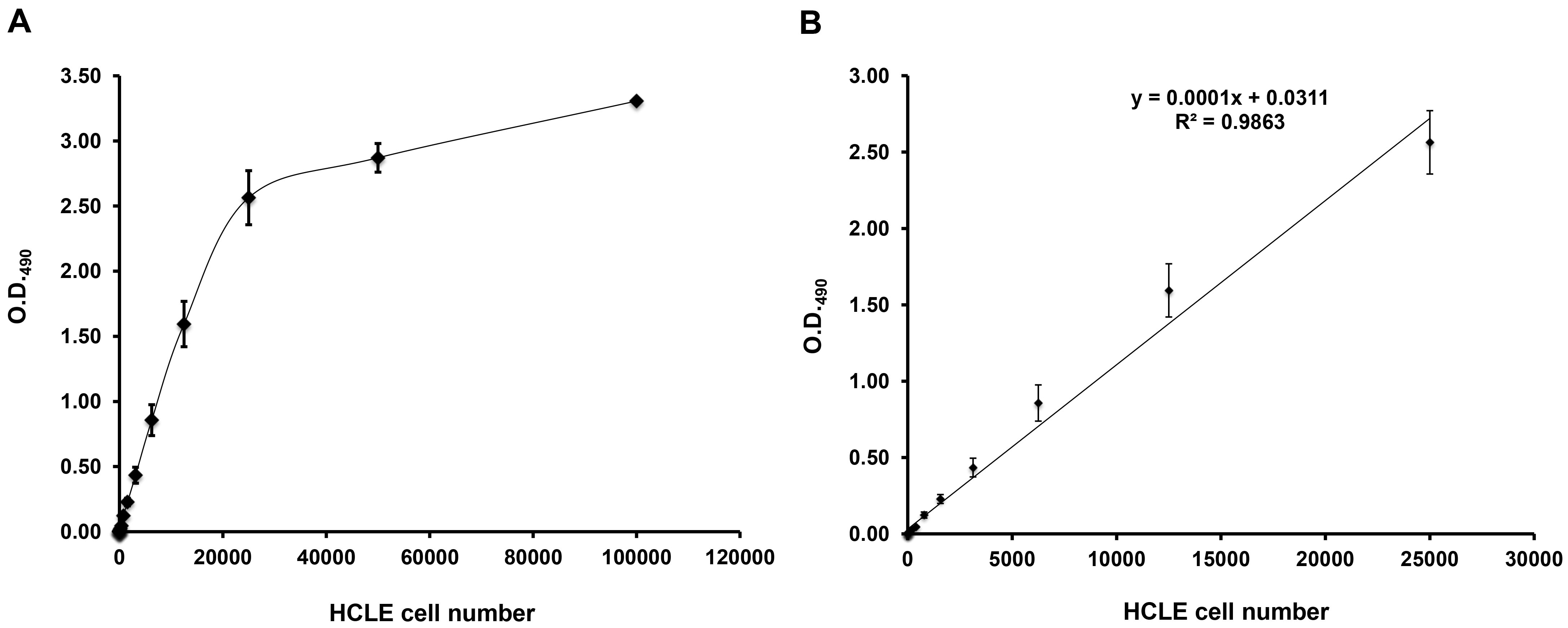

Supplement: Figure S1 [file sph001162015sf1.doc]
